# Intrinsic structural design of graphite anodes for fast-charging lithium-ion batteries: strategies, trade-offs, and perspectives

**DOI:** 10.3389/fchem.2026.1869263

**Published:** 2026-07-27

**Authors:** Haotian Wu, Wenyue Si, Peikai Zhang, Shengjun Ji, Bangsheng Yin

**Affiliations:** 1 College of Mechanical Engineering, Shandong Huayu University of Technology, Dezhou, Shandong, China; 2 Faculty of Mechanical and Automotive Engineering Technology, Universiti Malaysia Pahang, Pekan, Malaysia

**Keywords:** defect engineering, fast-charging lithium-ion batteries, graphite anodes, intrinsic structural design, Li^+^ diffusion kinetics

## Abstract

With the increasing demand for rapid energy replenishment in electric vehicles and portable electronics, fast-charging lithium-ion batteries require graphite anodes with improved Li^+^ transport kinetics while maintaining high energy density and long-term stability. Graphite remains the dominant commercial anode material, but its layered structure leads to anisotropic Li^+^ diffusion, limited edge-plane intercalation sites, concentration polarization, and lithium plating risk under high-rate charging. This mini-review focuses specifically on intrinsic structural design of graphite anodes, rather than electrolyte, artificial SEI, or charging-protocol optimization. Interlayer spacing regulation, porous structure construction, particle size/morphology optimization, and defect/edge-site engineering are summarized within a structure–kinetics–trade-off framework. These strategies can shorten Li^+^ diffusion pathways, increase effective Li^+^ entry sites, reduce polarization, and improve high-rate capacity utilization. However, they may also increase specific surface area, reduce initial Coulombic efficiency, decrease compaction density, and impair structural stability. Finally, practical evaluation criteria, including full-cell validation, high areal loading, high compaction density, limited lithium inventory, and lithium plating diagnosis, are discussed to guide future intrinsic graphite design for practical fast-charging batteries.

## 1 Introduction

With the continuously increasing demand for rapid energy replenishment in electric vehicles and portable electronics, the development of fast-charging lithium-ion batteries with high energy density, long cycle life, and reliable safety performance has become an important direction in electrochemical energy storage research (Li et al., 2022; Liu et al., 2023). Graphite anodes remain the most widely used anode materials in commercial lithium-ion batteries owing to their low cost, relatively high theoretical specific capacity, low lithiation potential, and industrial maturity (Dong et al., 2025; Liu et al., 2023). However, under high-rate charging conditions, graphite anodes are limited by sluggish Li^+^ intercalation and solid-state diffusion kinetics, which can lead to inhomogeneous lithiation, intensified concentration polarization, and even lithium plating, thereby causing capacity degradation and safety risks (Cai et al., 2020; Dong et al., 2025; Li et al., 2022).

For fast-charging applications, improving graphite anode performance relies not only on electrolyte optimization and interfacial regulation, but also on intrinsic material structural design to enhance Li^+^ transport within graphite particles (Dong et al., 2025; Liu et al., 2023). Previous studies have shown that interlayer spacing regulation, porous structure construction, particle size and morphology optimization, and defect/edge-site engineering are important strategies for improving the fast-charging capability of graphite materials (Ding et al., 2024; Li et al., 2022; Liu et al., 2023). These approaches can shorten Li^+^ diffusion pathways, increase effective Li^+^ entry sites, and reduce polarization at different structural scales; however, they may also lead to increased specific surface area, decreased initial Coulombic efficiency, reduced compaction density, and impaired structural stability (Ding et al., 2024; Liu et al., 2023; Yi et al., 2024).

Based on this background, this mini review focuses on the influence of intrinsic structural design strategies of graphite materials on fast-charging performance. The mechanisms, performance advantages, and potential limitations of interlayer spacing regulation, porous structure construction, particle size/morphology optimization, and defect/edge-site engineering are systematically discussed. The remaining challenges and future directions for their practical application in high-energy-density fast-charging batteries are further analyzed.

Compared with recent comprehensive reviews on fast-charging anodes and graphite anode modification, this mini-review provides a more focused discussion of intrinsic graphite structural design. Electrolyte formulation, artificial SEI construction, charging protocols, and cell management strategies are not treated as independent topics here. Instead, this review emphasizes how graphite bulk structural parameters, including interlayer spacing, porosity, particle size/morphology, and defect/edge-site density, regulate Li^+^ diffusion pathways, lithiation homogeneity, polarization, and lithium plating tendency. By organizing these strategies within a structure–kinetics–trade-off framework, this review aims to clarify not only how intrinsic structural regulation improves high-rate performance, but also why excessive structural opening may compromise initial Coulombic efficiency, compaction density, volumetric energy density, and long-term stability.

More specifically, the uniqueness of this mini-review lies in three aspects. First, it narrows the discussion to intrinsic graphite structural parameters, rather than treating electrolyte engineering, artificial SEI construction, charging protocols, and battery management as parallel topics. Second, it reorganizes interlayer spacing, porosity, particle morphology, and defect/edge-site engineering according to their effects on Li^+^ entry pathways, solid-state diffusion length, lithiation homogeneity, and polarization. Third, it links material-level structural modification with practical evaluation criteria, including full-cell configuration, areal loading, compaction density, limited lithium inventory, cycling retention, and lithium plating diagnosis. This focused perspective aims to provide a bridge between graphite structural design and practical fast-charging validation.

In this review, the structure–kinetics–trade-off framework refers to a three-level analysis. The “structure” level includes intrinsic graphite parameters such as interlayer spacing, pore size/density, particle size and morphology, edge-site exposure, and defect density. The “kinetics” level refers to their influence on Li^+^ entry pathways, solid-state diffusion distance, interfacial reaction kinetics, electrode polarization, and lithium plating tendency. The “trade-off” level evaluates the accompanying penalties, including increased specific surface area, reduced initial Coulombic efficiency, excessive SEI formation, lower compaction density, reduced volumetric energy density, and possible structural instability. Where available, these trade-offs are discussed using practical metrics such as areal capacity, C-rate protocol, SOC target, capacity retention, full-cell configuration, and lithium plating diagnosis.

## 2 Intrinsic structure–kinetics relationship of graphite during fast charging

Graphite is a typical layered intercalation-type anode material. During the charging process, Li^+^ is intercalated into graphite interlayers to form Li_x_C_n_ intercalation compounds, with fully lithiated graphite corresponding to LiC_6_ and delivering a theoretical specific capacity of approximately 372 mA h g^-1^ (Ding et al., 2024; Yan et al., 2024). The layered structure of graphite provides high structural stability and advantages for commercial applications, but it also leads to pronounced anisotropy in Li^+^ transport (Ding et al., 2024; Li et al., 2022). Previous studies have shown that Li^+^ preferentially enters graphite interlayers through edge planes, whereas direct penetration through the carbon hexagonal rings of the basal plane involves a relatively high energy barrier (Li et al., 2022; Liu et al., 2019). Therefore, the effective Li^+^ entry sites of graphite are mainly concentrated at edge regions. When graphite particles are large, edge sites are insufficient, or particle orientation is unfavorable, Li^+^ must undergo long solid-state diffusion pathways, thereby limiting rapid lithiation at high rates (Ding et al., 2024; Liu et al., 2019).

Under fast-charging conditions, the substantially increased current input requires Li^+^ to be rapidly intercalated and transported into the interior of graphite particles within a short time. If Li^+^ diffusion in the graphite bulk is insufficient, a pronounced concentration gradient forms between the particle surface and interior, resulting in inhomogeneous lithiation and intensified anode polarization (Ding et al., 2024; Li et al., 2022). Because graphite has a low lithiation potential, excessive polarization may drive the graphite surface potential close to or even below the Li/Li^+^ deposition potential, thereby triggering lithium plating and causing active lithium loss, capacity fading, and potential safety risks (Ding et al., 2024; Li et al., 2022; Yan et al., 2024).

It should be noted that the apparent fast-charging behavior of graphite is not governed by solid-state diffusion alone (Dong et al., 2025; Weiss et al., 2021). During high-rate charging, Li^+^ transport in the electrolyte, Li^+^ desolvation and interfacial charge transfer at the graphite/electrolyte interface, Li^+^ migration through the SEI, solid-state diffusion within graphite particles, and ion transport through the porous electrode network are strongly coupled (Liang et al., 2025). In thin electrodes or small particles, interfacial kinetics and SEI impedance may become dominant, whereas in thick electrodes with high loading, electrolyte transport and electrode tortuosity can substantially intensify concentration polarization (Tu et al., 2023; Weiss et al., 2021). Lithium plating occurs when the combined kinetic limitations drive the graphite surface potential close to or below the Li/Li^+^ deposition potential (Dong et al., 2025; Liang et al., 2025). Therefore, intrinsic structural design should be interpreted as one key component of the overall fast-charging kinetic network, rather than as an isolated factor.

**FIGURE 1:**
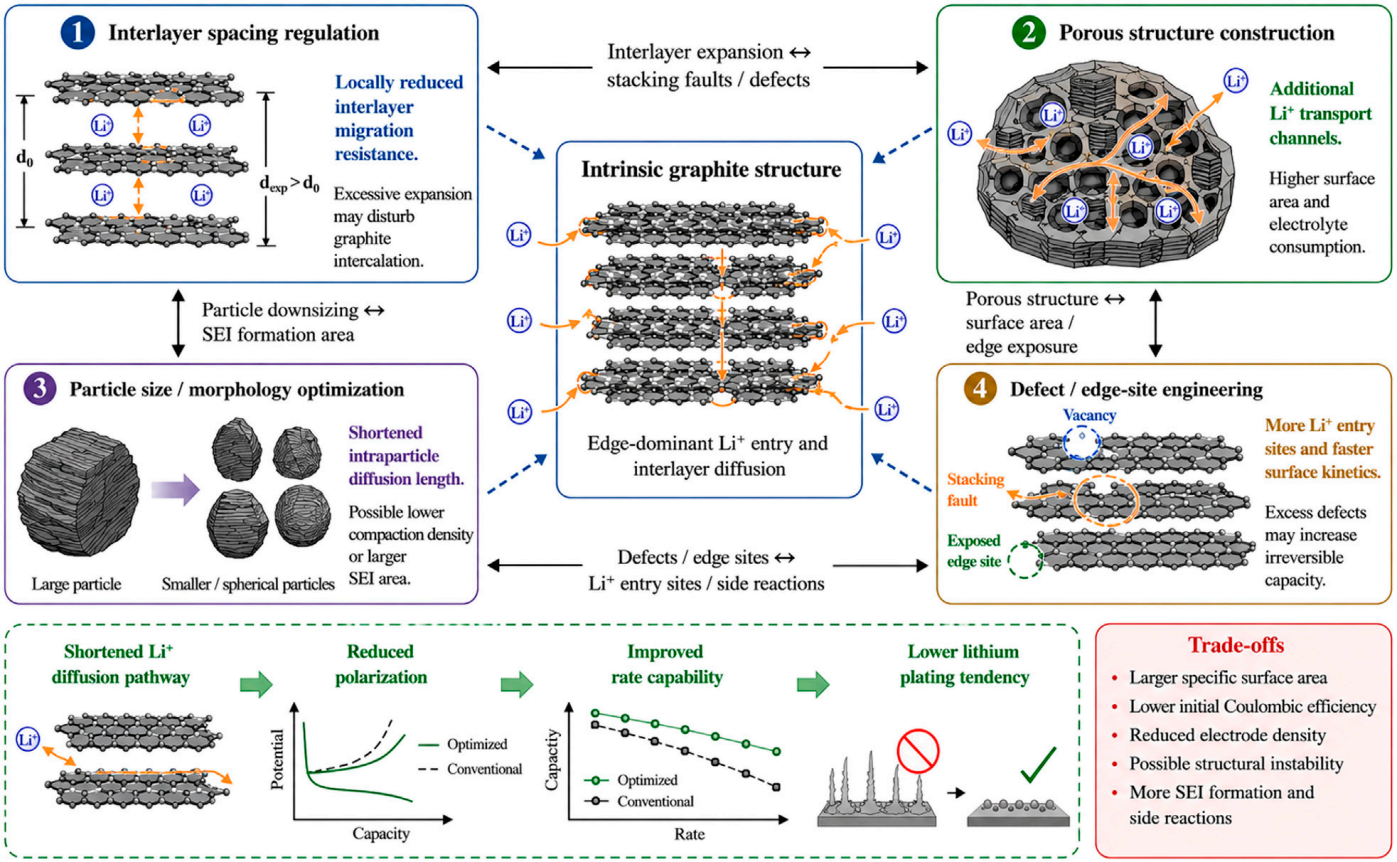
Coupled intrinsic structural parameters for fast-charging graphite anodes. Interlayer spacing regulation, porous structure construction, particle size/morphology optimization, and defect/edge-site engineering are not independent strategies, but are often coupled during graphite modification. These coupled structural parameters jointly affect Li^+^ transport pathways, polarization, rate capability, and lithium plating tendency, while introducing trade-offs in initial Coulombic efficiency, compaction density, volumetric energy density, and structural stability.

Therefore, there is a direct relationship between the intrinsic structure of graphite and its fast-charging kinetics: the layered structure determines the directionality of Li^+^ diffusion, edge-preferred intercalation limits the number of effective Li^+^ entry sites, and particle size, morphology, and defect state further affect diffusion length and reaction homogeneity (Cai et al., 2020; Liu et al., 2019). Based on this understanding, intrinsic structural design of graphite should focus on shortening Li^+^ diffusion pathways, increasing effective Li^+^ entry sites, reducing polarization, and maintaining structural stability, while avoiding the sacrifice of initial Coulombic efficiency, compaction density, and energy density caused by excessive structural opening (Cai et al., 2020; Li et al., 2022; Liu et al., 2019). The influence of intrinsic structural design strategies on the fast-charging performance of graphite is schematically illustrated in Figure 1.

## 3 Intrinsic structural design strategies of graphite materials

After clarifying the effects of the layered graphite structure, anisotropic Li^+^ diffusion, and edge-preferred intercalation on fast-charging kinetics, this section further discusses intrinsic structural regulation strategies for graphite materials. In general, intrinsic graphite design mainly focuses on interlayer spacing, pore structure, particle size/morphology, and defect/edge-site engineering, aiming to shorten Li^+^ diffusion pathways, reduce bulk transport resistance, increase effective Li^+^ entry sites, and improve lithiation homogeneity during high-rate charging.

### 3.1 Interlayer spacing regulation

The relatively small interlayer spacing of graphite is one of the key factors limiting rapid interlayer Li^+^ migration. The interlayer spacing of commercial graphite is approximately 0.335 nm, whereas that of fully lithiated LiC_6_ can reach approximately 0.370 nm. Therefore, expanding the graphite interlayer spacing within a reasonable range can help reduce steric hindrance during interlayer Li^+^ migration, increase the Li^+^ diffusion rate, and improve lithiation/delithiation kinetics under fast-charging conditions (Dong et al., 2025; Liu et al., 2023).

The expansion of graphite interlayer spacing can generally be achieved through chemical intercalation, oxidation treatment, and thermal exfoliation (Ding et al., 2024; Dong et al., 2025; Li et al., 2022). For example, Zou et al. prepared mildly expanded graphite through mild intercalation using an H_2_SO_4_–H_2_O_2_ system followed by thermal treatment. This method increased the average graphite interlayer spacing from 0.3358 nm to 0.3370 nm, while simultaneously introducing structural features such as pores, channels, and stacking faults. It should be emphasized that such a small change in the average interlayer spacing alone is unlikely to fully account for the reported electrochemical improvement. Rather, the enhanced performance should be understood as the combined result of slight interlayer expansion, local structural opening, stacking disorder, additional transport channels, and defect/edge-related Li^+^ storage sites. Therefore, mildly expanded graphite should not be interpreted simply as a uniformly expanded lattice, but as a structurally modified graphite in which local transport pathways and accessible reaction sites are simultaneously altered. These coupled structural changes can buffer lithiation/delithiation-induced structural variation and provide additional Li^+^ storage/transport sites, thereby improving reversible capacity and cycling stability (Ding et al., 2024; Zou et al., 2009). Related reviews have also indicated that H_2_SO_4_ and H_2_O_2_ can act as intercalants to enter graphite interlayers, and subsequent thermal treatment removes the intercalants to form a mildly expanded structure; meanwhile, surface defects generated during oxidation can provide additional Li^+^ storage sites and promote Li^+^ diffusion (Ding et al., 2024).

In addition to overall interlayer expansion, local interlayer spacing regulation is also an important approach for improving fast-charging performance. In particular, expanding the interlayer spacing near graphite edge regions can enhance Li^+^ intercalation/deintercalation kinetics (Dong et al., 2025; Park et al., 2012). Park et al. achieved edge-selective functionalization, in which functional groups served as “molecular wedges” to locally enlarge the interlayer spacing at graphite edges while maintaining the overall structural integrity of graphite. This edge-expanded structure facilitated Li^+^ entry, and the resulting material showed an increase in capacity from 110 mAh g^-1^ to 190 mAh g^-1^ at 50 °C (Park et al., 2012). Du et al. constructed edge-activated graphite through solution treatment and calcination. Benefiting from additional Li^+^ storage sites and an expanded edge structure, the material exhibited enhanced rate performance, delivering 150.3 mA h g^-1^ at 10 °C and maintaining a capacity retention of 96.05% after 700 cycles at 5 °C (Du et al., 2022).

It should be noted that a larger interlayer spacing is not always preferable. If the interlayer spacing is excessively enlarged, the material structure may approach stacked monolayer graphene, and its lithiation mechanism may deviate from the conventional graphite intercalation reaction (Li et al., 2022). Therefore, interlayer spacing regulation should aim to reduce Li^+^ diffusion resistance while maintaining the characteristic intercalation reaction of graphite, rather than excessively disrupting the layered structure. In addition, interlayer expansion is often accompanied by oxidation, defect formation, and changes in specific surface area, which may affect the initial Coulombic efficiency and interfacial side reactions. Therefore, the quantitative relationship among interlayer spacing, crystal structure, and Li^+^ diffusion behavior still needs to be further clarified.

### 3.2 Porous structure construction

Constructing porous structures is another important strategy for shortening Li^+^ diffusion pathways and increasing active lithiation sites. Because graphite typically exhibits edge-preferred Li^+^ intercalation, Li^+^ must diffuse from the particle edges toward the interior. Introducing pores into graphite particles can provide more channels for Li^+^ to access the interlayers, thereby improving capacity utilization at high rates (Li et al., 2022; Liu et al., 2023).

KOH etching is a commonly used method for constructing porous graphite. Related reviews have indicated that KOH etching can generate porous channel structures on the graphite surface, which not only allow Li^+^ intercalation from edge planes but also provide transport pathways for Li^+^ entering graphite from the basal-plane direction, while increasing accessible Li^+^ adsorption/intercalation sites (Cheng et al., 2015; Li et al., 2022). Cheng et al. reported a multichannel graphite structure with an average pore size of approximately 10 nm. This porous structure shortened the Li^+^ intercalation pathway in graphite and thus improved charge–discharge performance (Cheng et al., 2015; Cheng and Zhang, 2018). Weiss et al. also noted that KOH solution etching, followed by drying and annealing under a nitrogen atmosphere, can form nanoscale pores in graphite, and the etched graphite exhibits higher specific capacity than pristine graphite at high current densities (Weiss et al., 2021). In addition, nickel-catalyzed steam gasification has also been used to construct porous graphite structures, leading to improved high-rate cycling stability and reversible capacity (Deng and Zhou, 2016).

However, porous structure design requires careful control. Li et al. pointed out that controlling the etching temperature is important; only under mild conditions can the pore structure be confined to the nanoscale while avoiding obvious structural damage and irreversible capacity loss (Li et al., 2022). Although porous graphite is beneficial for fast-charging kinetics, excessive pore formation may also increase the specific surface area, accelerate electrolyte consumption, decrease the initial Coulombic efficiency, and reduce compaction density and volumetric energy density (Dong et al., 2025). Therefore, porous graphite design should not simply pursue higher porosity, but should instead balance pore size, pore density, specific surface area, compaction density, and side reactions.

### 3.3 Particle size regulation and spherical morphology design

The particle size of graphite directly affects the Li^+^ diffusion length, number of active sites, specific surface area, compaction density, and interfacial side reactions. Larger graphite particles generally have a lower specific surface area, resulting in fewer side reactions with the electrolyte and higher initial Coulombic efficiency; however, their longer Li^+^ diffusion pathways and limited active sites/intercalation channels restrict high-rate performance. In contrast, reducing graphite particle size can shorten the intraparticle Li^+^ diffusion distance, decrease diffusion resistance, and improve active material utilization at high rates (Liu et al., 2023; Wang et al., 2023; Weiss et al., 2021).

Weiss et al. analyzed the influence of particle size on fast-charging performance from a materials perspective. Larger graphite particles typically exhibit lower charging capacity at high rates, mainly owing to sluggish solid-state Li^+^ diffusion and longer diffusion pathways. When comparing flake graphite with different particle sizes, smaller active materials showed easier and more complete lithiation/delithiation, whereas 44 μm particles could only undergo partial lithiation at high rates (Rangom et al., 2021; Weiss et al., 2021). Therefore, from the perspective of Li^+^ diffusion, particle size reduction is beneficial for fast charging; however, excessively small particles also markedly increase the specific surface area, leading to greater capacity loss during formation and enhanced side reactions with the electrolyte (Cai et al., 2020; Weiss et al., 2021).

Spherical morphology design is also a commonly used strategy for morphology regulation. Related reviews have indicated that reducing graphite particle size or adopting a spherical morphology can shorten Li^+^ diffusion pathways and thereby improve rate performance (Liu et al., 2023; Ruggeri et al., 2022). Spherical graphite exhibits good processability and packing characteristics in commercial anodes, which helps improve electrode compaction density and structural uniformity (Martin et al., 2023; Ruggeri et al., 2022). However, in fast-charging design, it remains necessary to consider whether sufficient Li^+^ diffusion channels exist inside spherical particles, whether particle orientation causes localized diffusion limitations, and whether excessive densification hinders electrolyte wetting and ion transport. Therefore, the key to particle size and spherical morphology design is not simply to pursue “smaller” or “more spherical” particles, but to construct graphite particles that integrate short diffusion pathways, high compaction density, suppressed side reactions, and stable SEI formation (Shi et al., 2019).

### 3.4 Defect and edge-site engineering

Defect and edge-site engineering is an important complementary strategy for enhancing the fast-charging kinetics of graphite. An ideal graphite lattice is highly ordered and structurally intact; however, this also means that Li^+^ intercalation sites are limited and mainly concentrated at edge regions. The appropriate introduction of edge vacancies, dislocations, stacking faults, or surface defects can increase Li^+^ reaction sites and transport pathways, thereby improving surface reaction kinetics and bulk lithiation behavior (Dong et al., 2025; Liu et al., 2023). Related reviews have classified defects into point defects, line defects, and planar defects, and have indicated that surface defects such as edge vacancies, interstitial atoms, dislocations, and layered defects generally increase the surface area and provide additional ionic reaction sites, thereby promoting surface and bulk lithiation kinetics (Dong et al., 2025).

From the perspective of lithium storage mechanisms, defect structures may also introduce pseudocapacitive behavior. Materials with abundant defects are more likely to exhibit pseudocapacitive lithium storage, which usually occurs at or near the electrode surface and proceeds rapidly, thus benefiting high-rate performance (Liu et al., 2023; Wang et al., 2022). Wang et al. prepared activated graphite (AG) by thermally treating natural graphite in Ar atmospheres containing different CO_2_ concentrations. Compared with pristine graphite with an intact lattice and sluggish kinetics, the near-edge defects introduced into AG significantly enhanced interfacial reactions and induced a pseudocapacitive lithium storage mechanism. The AG anode delivered 209 mAh g^-1^ at 10 °C, and the assembled LFP//AG full cell reached 82% and 96% SOC within 6 and 15 min, respectively (Wang et al., 2022).

Experimentally, pseudocapacitive lithium storage is usually distinguished from diffusion-controlled intercalation by analyzing the dependence of current response on scan rate in cyclic voltammetry. The relationship can be expressed as i $=av^{b}$ , where *b* values close to 0.5 indicate diffusion-controlled behavior, whereas *b* values approaching 1.0 suggest surface-controlled or capacitive behavior. In addition, the current response can be separated into capacitive and diffusion-controlled contributions using $i\left(V\right)=k_{1}v+k_{2}v^{1/2}$ . For defect-engineered graphite, an increased capacitive contribution at high scan rates, together with reduced polarization and improved high-rate capacity, supports the presence of fast surface or near-surface lithium storage. Nevertheless, this contribution should be interpreted as complementary to graphite intercalation rather than a complete replacement of the conventional staged intercalation mechanism.

Mechanical treatment is also a common method for introducing defects and edge sites. For example, ball milling can increase graphite edge sites, thereby facilitating Li^+^ transport and improving rate performance (Dong et al., 2025; Wang et al., 1998). Ball milling combined with mild oxidation can also increase exposed edges and anchoring sites for subsequent modification. However, defect regulation also has clear limitations. Excessive defects can increase the specific surface area, promote electrolyte side reactions and excessive SEI growth, and consequently reduce the initial Coulombic efficiency and increase irreversible capacity loss (Dong et al., 2025; Lee et al., 2020; Liu et al., 2023). Therefore, defect engineering should emphasize “moderate” and “controllable” regulation: it should provide additional Li^+^ channels and reaction sites while avoiding damage to the graphite framework and interfacial stability.

### 3.5 Synergistic optimization of intrinsic structural parameters

The above intrinsic design strategies are not independent of one another, but are often coupled during practical modification processes. Expansion of interlayer spacing is usually accompanied by stacking faults, pore formation, and changes in surface defects (Liu et al., 2023; Zou et al., 2009). Porous structures can provide additional Li^+^ transport channels, but they also increase the electrode/electrolyte contact area (Cheng et al., 2015; Dong et al., 2025). Particle size reduction shortens intraparticle diffusion pathways, but may increase the surface area available for SEI formation (Cai et al., 2020; Wang et al., 2023). Defects and edge sites can enhance reaction kinetics, whereas excessive defects may weaken lattice integrity and increase irreversible capacity loss (Li et al., 2022; Wang et al., 2022).

Therefore, intrinsic graphite design should not pursue the maximization of a single structural parameter. Instead, interlayer spacing, pore structure, particle size/morphology, and defect density should be optimized in a coordinated manner to balance Li^+^ diffusion kinetics, structural stability, initial Coulombic efficiency, compaction density, and volumetric energy density (Cai et al., 2020; Dong et al., 2025; Li et al., 2022). For fast-charging graphite anodes, the ideal intrinsic structure should provide shortened Li^+^ transport pathways, sufficient effective Li^+^ entry sites, a controllable specific surface area, and a stable graphitic framework. The effects and potential limitations of different intrinsic graphite design strategies on fast-charging performance are summarized in Table 1. The coupled relationships among these intrinsic structural parameters are schematically illustrated in Figure 1.

**TABLE 1:** Summary of intrinsic structural regulation strategies, fast-charging mechanisms, and potential limitations of graphite materials.

Strategy	Structural regulation	Fast-charging effect	Main trade-off
Interlayer spacing regulation	Intercalation, oxidation, thermal exfoliation, edge expansion	Reduced Li^+^ interlayer diffusion resistance	Structural disorder and side reactions
Porous structure construction	KOH etching, catalytic gasification, multichannel pores	Additional Li^+^ pathways and shortened diffusion distance	Larger surface area and lower compaction density
Particle size regulation	Particle downsizing and size distribution optimization	Shorter solid-state Li^+^ diffusion length	Lower initial coulombic efficiency
Spherical morphology design	Spherical/near-spherical particles and improved packing	Better processability and electrode compaction	Limited electrolyte wetting and internal Li^+^ transport
Defect/edge-site engineering	Oxidation, ball milling, thermal treatment, vacancies/stacking faults	More Li^+^ entrances and faster surface kinetics	Lattice damage and increased irreversible capacity

## 4 Practical evaluation of intrinsically modified graphite for fast charging

Intrinsic structural modification of graphite, including interlayer regulation, pore construction, particle morphology optimization, and defect/edge-site engineering, can effectively improve material-level Li^+^ transport kinetics (Dong et al., 2025; Liang et al., 2025; Weiss et al., 2021). However, improved rate capability in Li||graphite half cells does not necessarily indicate practical fast-charging capability in commercial lithium-ion batteries (Tu et al., 2023; Weiss et al., 2021). Fast charging at the cell level is governed by coupled processes, including Li^+^ transport in electrolyte-filled porous electrodes, desolvation and charge transfer at the graphite/electrolyte interface, Li^+^ migration through the SEI, solid-state diffusion inside graphite particles, electrode tortuosity, heat generation, and lithium plating tendency (Lee et al., 2021; Weiss et al., 2021). Therefore, intrinsically modified graphite should be evaluated not only by high-rate specific capacity, but also by its ability to maintain high capacity utilization, low polarization, stable interfacial impedance, and suppressed lithium plating under practical full-cell conditions (Jeong et al., 2024; Lee et al., 2021; Tu et al., 2023).

### 4.1 From high-rate half cells to practical full-cell fast charging

Li||graphite half cells are useful for identifying material-level kinetic improvements because they provide a simple configuration to evaluate Li^+^ intercalation behavior, rate capability, diffusion resistance, and polarization (Liang et al., 2025; Weiss et al., 2021). Nevertheless, they have clear limitations when used to claim practical fast-charging performance. In half cells, the lithium metal counter electrode provides an excess lithium reservoir, which can mask irreversible Li^+^ loss caused by increased surface area, defect-rich sites, continuous SEI growth, or electrolyte side reactions (Cai et al., 2020; Weiss et al., 2021). In addition, half-cell tests are often conducted using low-mass-loading electrodes, sufficient electrolyte, thin electrode layers, and relatively mild temperature conditions, which may underestimate ion-transport limitations and overestimate the fast-charging capability of graphite materials (Liang et al., 2025).

By contrast, practical full-cell fast charging requires graphite anodes to operate under limited lithium inventory, matched cathode/anode capacity, realistic N/P ratio, high areal loading, high compaction density, and finite electrolyte amount (Tu et al., 2023). Under these conditions, Li^+^ transport through the porous electrode and electrolyte becomes more difficult, while local concentration gradients and overpotential increase rapidly during high-current charging (Weiss et al., 2021). Previous materials-oriented analyses have emphasized that fast-charging performance is limited not only by lithium diffusion in active materials, but also by electrolyte-phase transport, interfacial charge transfer, electrode-level polarization, and lithium plating on graphite anodes (Tu et al., 2023).

Recent full-cell studies illustrate why practical validation is essential. Defect-engineered activated graphite delivered 209 mAh g^-1^ at 10 °C in half cells, and the corresponding LiFePO_4_||activated graphite full cell reached 82% and 96% SOC within 6 and 15 min, respectively (Wang et al., 2022). Soft-carbon-filled expanded graphite was further tested in an EGC||LiNi_0.8_Co_0.1_Mn_0.1_O_2_ full cell and showed near 20 min charging to 80% SOC, while the soft carbon filling was used to stabilize expanded graphite layer pores against structural collapse during fast charging (Quan et al., 2024). Vertical graphene sheets grown on natural graphite enabled a LiFePO_4_ full cell to deliver 312.1 Wh kg^-1^ at 4C with about 10 min charging, showing that fast Li^+^ transport pathways can translate into full-cell fast-charging performance when energy density is also considered (Mu et al., 2021).

However, not all graphite modification studies provide full-cell-level evidence. Some studies report excellent high-rate capacity or cycling stability mainly at the material or half-cell level (Liang et al., 2025). For example, multichannel graphite showed 83% capacity retention for 6C charging and 73% for 10C charging, as well as 85% capacity retention after 3000 cycles at 6C, but practical full-cell parameters such as areal loading, electrode density, electrolyte amount, and N/P ratio were not fully reflected in the available report (Cheng and Zhang, 2018). Therefore, high-rate half-cell performance should be regarded as an initial screening metric rather than definitive evidence of practical fast charging (Tu et al., 2023; Weiss et al., 2021).

### 4.2 Key metrics for evaluating fast-charging graphite anodes

To make different intrinsic graphite modification strategies comparable, fast-charging performance should be evaluated using standardized and practically relevant metrics (Dong et al., 2025; Weiss et al., 2021). First, electrode parameters should be clearly reported, including active-material mass loading, areal capacity, electrode thickness, porosity, compaction density, calendaring pressure, binder/conductive carbon ratio, and electrode geometry. These parameters strongly influence ion transport, tortuosity, local polarization, and heat generation during fast charging (Gottschalk et al., 2024; Weiss et al., 2021).

Second, cell configuration and testing protocol should be specified. Important information includes whether the test is conducted in a half cell, coin-type full cell, pouch cell, or larger-format cell; the cathode material; N/P ratio; electrolyte composition and dosage; separator type; voltage window; test temperature; charging mode; C-rate definition; and time required to reach 80% SOC (Tu et al., 2023; Weiss et al., 2021). Without these details, it is difficult to distinguish intrinsic material advantages from favorable testing conditions. The Li_3_P-based crystalline SEI work provides a useful benchmark because its pouch-cell evaluation reported fast charging at 6C and 10C, capacity retention over long-term cycling, and practical cell parameters such as mass loading, electrolyte dosage, and voltage window (Tu et al., 2023).

Third, electrochemical evaluation should include both kinetic and durability indicators. High-rate specific capacity is important, but it should be considered together with initial Coulombic efficiency, average Coulombic efficiency, capacity retention, polarization evolution, voltage hysteresis, charge-transfer resistance, SEI impedance, and evidence of lithium plating (Lee et al., 2021; Tu et al., 2023). For example, MoO_x_–MoP_x_/graphite paired with a LiNi_0.6_Co_0.2_Mn_0.2_O_2_ cathode showed less than 10 min charging for 80% capacity and stable cycling over 300 cycles without signs of lithium plating, providing a useful benchmark for validating modified graphite under full-cell conditions (Lee et al., 2021). Similarly, Co_2_P-modified natural graphite enabled a full cell to reach 80% SOC within 16.1 min and maintain stable cycling over 300 cycles, highlighting the importance of full-cell fast-charging evaluation (Jeong et al., 2024).

Fourth, practical evaluation should include both gravimetric and volumetric performance. A modified graphite anode with high specific capacity at high rate may still be unsuitable for practical fast-charging batteries if the electrode density, areal capacity, or full-cell energy density is substantially reduced (Dong et al., 2025; Liang et al., 2025). Therefore, areal capacity, volumetric capacity, electrode density, and full-cell energy density should be reported together with rate capability (Gottschalk et al., 2024). Spherical graphite and multilayer anode studies further demonstrate that particle size distribution, pore network, ionic resistance, and electrode architecture strongly affect lithium-ion cell performance, rather than only the intrinsic properties of individual graphite particles (Gottschalk et al., 2024).

Representative graphite-based fast-charging studies and their reported practical evaluation metrics are summarized in Table 2. This comparison highlights that high-rate specific capacity alone is insufficient to evaluate fast-charging capability. Instead, cell configuration, charging protocol, SOC target, full-cell validation, electrode-level information, cycling stability, and lithium plating diagnosis should be considered together (Lee et al., 2021; Weiss et al., 2021).

**TABLE 2:** Representative graphite-based fast-charging studies and verified practical electrochemical parameters.

Strategy/role	Graphite system	Cell format	Verified reported parameters	Verified fast-charging/cycling result	Interpretation
Defect engineering	CO_2_-activated graphite/activated graphite (AG)	Li||AG half cell; LiFePO^4^||AG full cell	Natural graphite was treated under CO_2_-containing atmosphere at 850 °C; pseudocapacitive lithium storage was introduced by intrinsic defect engineering	AG delivered 209 mAh g^-1^ at 10C in half cell; LiFePO^4^||AG full cell reached 82% and 96% SOC within 6 and 15 min, respectively; full cell retained 87.3% capacity after 50 cycles at 8C	Demonstrates that defect-enhanced kinetics can translate into full-cell fast-charging behavior
Expanded graphite stabilization	Soft-carbon-filled expanded graphite (EGC)	Li||EGC half cell; EGC||NCM811 full cell	Half-cell electrode mass loading: 1.0–1.2 mg cm^-2^; CV potential window: 0.01–2.0 V vs. Li/Li+; EGC||NCM811 full cell tested with N/P ratio of 1.1; 1C@NCM811 = 170 mA g^-1^; rate test used different charging rates from 0.1C to 5C with 0.2C discharge	EGC maintained 305 mAh g^-1^ for over 600 cycles at 1C; EGC||NCM811 full cell retained 70.59% capacity after 200 cycles at 170 mA g^-1^; at 3C, the full cell delivered 160 mA h g^-1^ initially and 126 mA h g^-1^ after 50 cycles; near 20 min charging to 80% SOC was reported	Soft-carbon filling stabilizes expanded graphite layer pores and supports fast-charging durability
Vertical Li^+^ transport pathways	Vertical graphene sheets on graphite (VGSs/graphite)	VGSs/graphite||LiFePO^4^ full cell	Energy-density calculation used electrode area of 0.95 cm^2^; reported active-material thicknesses: Graphite 68.1 μm, VGSs/graphite 52.1 μm, LiFePO^4^ 88.2 μm	VGSs/graphite full cell delivered 433.9, 423.6, 400.9, 378.4, 345.1, and 312.1 Wh kg^-1^ at 0.2, 0.5, 1, 2, 3, and 4C, respectively; charging time was about 10 min at 4C	Links vertical Li^+^ transport pathways with high-rate full-cell energy-density retention
Multichannel pore construction	Air-oxidized multichannel graphite	Full-cell rate evaluation; symmetric-cell EIS	Natural graphite CGB-20, 20 μm; treatment at 650, 750, or 850 °C in dry air for 1 h followed by N^2^ treatment; full-cell voltage range: 2.5–4.2 V; rate test charged at 0.1–10C and discharged at 0.1C; cycling used 6C CC charge and 0.1C CC discharge; symmetric-cell EIS was measured at SOC50% from −20 °C to 20 °C	Air graphite 850 showed 83% capacity retention at 6C and 73% at 10C; after 3000 cycles at 6C, capacity retention remained almost 90%; charge-transfer resistance at −20 °C decreased from 1024 Ω for pristine graphite to 330 Ω for air graphite 850; activation energy decreased from 65.9 to 56.3 kJ mol^-1^	Strong material/electrode-level evidence that multichannel structures reduce transport resistance and improve high-rate durability
Particle/electrode structuring	Spherical graphite multilayer anode	Lithium-ion full cells	Spherical graphite particles with D50 = 18 μm and 11 μm; all anodes had areal capacity of 4 mAh cm^-2^, with 2 mAh cm^-2^ for each layer in two-layer anodes; electrolyte: 150 μL LP57, 1 M LiPF^6^ in EC/EMC 3:7 w/w with 2 wt% VC; test temperature: 20 °C; charge-rate test from 0.2C to 3C; discharge fixed at 0.2C; cycling window: 2.9–4.2 V; ageing test used 50 cycles at 1C	Two-layer anodes reduced ionic resistance and improved ageing behavior compared with one-layer references; one-layer anodes lost approximately 12 mAh g^-1^ after ageing, corresponding to about 8%–10% of initial capacity	Highlights electrode-scale transport, pore-network design, and particle-size distribution as practical fast-charging factors
Practical SEI benchmark	Li_3_P-based crystalline SEI on P-S-graphite	NCM622||P-S-graphite pouch cell	P-S-graphite areal capacity: ∼2.3 mAh cm^-2^; pouch-cell voltage range: 2.9–4.25 V; CC–CV charging protocol; CC charging rates from 0.2C to 10C; discharge fixed at 0.2C; N/P ratio: ∼1.1; pouch-cell capacity: ∼1 Ah	Pouch cell charged 97.0%, 91.2%, 86.2%, and 80.0% capacity within 15, 10, 7.5, and 6 min at 4C, 6C, 8C, and 10C, respectively; capacity retention was 82.9% after 2000 cycles at 6C	Included as a practical SEI/interfacial benchmark for full-cell fast-charging evaluation, not as a purely intrinsic bulk-structure modification
Surface-promoter benchmark	MoO_x_–MoP_x_/graphite, Mo-CP/graphite	NCM622||Mo-CP/graphite full cell	Mo-CP loading: 1–2 wt%; MoOx nanolayer thickness: ∼10 nm before phosphidation; graphite surface modified with cooperative MoO_x_–MoP_x_ promoter	Full cell with NCM622 cathode and Mo-CP/graphite anode showed <10 min charging for 80% capacity and stable cycling over 300 cycles under fast-charging conditions	Included as a surface-promoter/interfacial benchmark because it reports full-cell fast charging and Li plating suppression
Interfacial-phosphide benchmark	Co_2_P-modified natural graphite, Co_2_P@NG	Li||Co_2_P@NG half cell; NCM||Co_2_P@NG full cell	Anode electrode composition: Active material/SBR/CMC = 96:2:2; anode loading: 6.5 mg cm^-2^; electrode density: 1.5 g cm^-3^; half-cell electrolyte: 1 M LiPF^6^ in EC/DMC = 3:7; half-cell voltage window: 0.01–1.5 V vs. Li/Li^+^; full cell used commercial NCM cathode	Co^2^P@NG full cell showed charging time of 16.1 min–80% SOC and cycling over 300 cycles; half-cell initial charge/discharge capacities were 382.7/353.5 mA h g^-1^ with ICE of 92.2%	Included as an interfacial-phosphide benchmark; it provides SOC-based full-cell fast-charging evidence and Li plating suppression mechanism

AG, activated graphite, EGC; soft-carbon-filled expanded graphite, VGSs; vertical graphene sheets, NCM811; LiNi_0.8_Co_0.1_Mn_0.1_O_2_, NCM622; LiNi_0.6_Co_0.2_Mn_0.2_O_2_, NCM; nickel–cobalt–manganese oxide cathode as reported in the cited study, SEI; solid–electrolyte interphase, SOC; state of charge, N/P; negative-to-positive electrode capacity ratio, ICE; initial Coulombic efficiency. Only parameters directly identified from the cited studies are listed. Interfacial modification studies are included only as practical fast-charging benchmarks because they provide full-cell metrics and lithium plating diagnosis, they should not be interpreted as purely intrinsic bulk-structure modifications.

## 5 Challenges and perspectives

Although intrinsic structural regulation provides an effective route for improving the fast-charging kinetics of graphite anodes, several key challenges remain before these strategies can be translated into practical high-energy-density lithium-ion batteries (Dong et al., 2025; Liu et al., 2023; Weiss et al., 2021). Future research should move beyond the pursuit of high-rate capacity alone and focus on establishing quantitative relationships among structural parameters, kinetic behavior, electrode-level transport, and cell-level stability (Weiss et al., 2021; Yan et al., 2024).

First, quantitative structure–kinetics relationships should be established. Current studies have shown that interlayer spacing regulation, pore construction, particle size optimization, and defect/edge-site engineering can improve Li^+^ transport kinetics, but the optimal ranges of these parameters remain unclear (Dong et al., 2025; Liang et al., 2025; Liu et al., 2023). Future work should correlate interlayer spacing, pore size, pore density, particle size distribution, defect density, and edge-site exposure with measurable kinetic parameters, such as Li^+^ diffusion coefficients, charge-transfer resistance, SEI impedance, polarization evolution, and lithium plating tendency (Weiss et al., 2021; Yan et al., 2024). Such quantitative correlations would help shift graphite design from empirical modification toward predictive structural optimization.

Second, scalable and controllable modification methods remain essential. Chemical expansion, KOH etching, catalytic gasification, ball milling, and controlled atmosphere activation can improve fast-charging behavior, but these methods may introduce challenges related to cost, environmental impact, structural uniformity, batch-to-batch consistency, and compatibility with industrial electrode processing (Dong et al., 2025; Liang et al., 2025). Therefore, future intrinsic graphite modification should prioritize mild, reproducible, environmentally acceptable, and scalable processes that can preserve graphitic structural stability while improving Li^+^ transport kinetics (Dong et al., 2025; Liu et al., 2023).

Third, standardized full-cell evaluation should be further strengthened. Although the need for full-cell validation has been increasingly recognized, reporting standards still vary widely across different studies (Tu et al., 2023; Weiss et al., 2021). Future studies should provide complete information on electrode loading, compaction density, electrolyte amount, N/P ratio, voltage window, test temperature, charging protocol, SOC target, cycling retention, and lithium plating diagnosis (Tu et al., 2023; Yan et al., 2024). Such standardized reporting would enable a more reliable comparison among different intrinsic graphite design strategies and help identify which modifications are truly compatible with high-energy-density fast-charging batteries.

Finally, diagnosis and lifetime prediction should be integrated into the development of fast-charging graphite anodes. During repeated fast charging, graphite anodes may suffer from lithium plating, impedance growth, SEI reconstruction, local overpotential accumulation, and structural degradation (Tu et al., 2023; Wang et al., 2022). Data-driven failure detection, degradation monitoring, and prognosis and health management concepts may provide useful tools for identifying early-stage abnormal behavior before severe capacity loss or safety failure occurs (Liu, 2023; Sadegh Kouhestani et al., 2022). Therefore, future fast-charging graphite research should combine intrinsic structural design, practical full-cell testing, and battery-level diagnostic methods to achieve fast charging, long cycle life, and reliable safety simultaneously.

Overall, the future development of intrinsically modified graphite anodes should focus on the integration of quantitative structural design, scalable processing, standardized full-cell validation, and advanced degradation diagnosis. Only by linking material-level structure regulation with electrode- and cell-level performance requirements can graphite anodes better meet the demands of practical high-energy-density fast-charging lithium-ion batteries (Weiss et al., 2021; Yan et al., 2024).

## 6 Conclusion

In summary, intrinsic structural design of graphite materials represents an important approach for improving fast-charging performance. Interlayer spacing regulation, porous structure construction, particle size/morphology optimization, and defect/edge-site engineering can improve Li^+^ transport kinetics, reduce polarization, and enhance high-rate capacity utilization at different structural scales. However, these strategies are also accompanied by trade-offs in initial Coulombic efficiency, compaction density, structural stability, and energy density. In addition, high-rate half-cell performance should not be directly regarded as practical fast-charging capability; full-cell validation under realistic loading, compaction density, limited lithium inventory, and lithium plating diagnosis is essential. Therefore, future efforts should focus on multiparameter synergistic optimization and standardized full-cell evaluation to achieve a balance among fast-charging capability, cycling stability, energy density, safety, and scalable manufacturing.
